# Novel Trends in the Development of Surfactant-Based Hydraulic Fracturing Fluids: A Review

**DOI:** 10.3390/gels7040258

**Published:** 2021-12-12

**Authors:** Andrey V. Shibaev, Andrei A. Osiptsov, Olga E. Philippova

**Affiliations:** 1 Physics Department, Moscow State University, 119991 Moscow, Russia; phil@polly.phys.msu.ru; 2 Skolkovo Institute of Science and Technology (Skoltech), 121205 Moscow, Russia; a.Osiptsov@skoltech.ru

**Keywords:** viscoelastic surfactants, wormlike micelles, gels, viscoelasticity, oil recovery, hydraulic fracturing, clean fracturing fluids, responsiveness to hydrocarbons, oligomeric surfactants, nanoparticle/VES fluids, polymer/VES fluids

## Abstract

Viscoelastic surfactants (VES) are amphiphilic molecules which self-assemble into long polymer-like aggregates—wormlike micelles. Such micellar chains form an entangled network, imparting high viscosity and viscoelasticity to aqueous solutions. VES are currently attracting great attention as the main components of clean hydraulic fracturing fluids used for enhanced oil recovery (EOR). Fracturing fluids consist of proppant particles suspended in a viscoelastic medium. They are pumped into a wellbore under high pressure to create fractures, through which the oil can flow into the well. Polymer gels have been used most often for fracturing operations; however, VES solutions are advantageous as they usually require no breakers other than reservoir hydrocarbons to be cleaned from the well. Many attempts have recently been made to improve the viscoelastic properties, temperature, and salt resistance of VES fluids to make them a cost-effective alternative to polymer gels. This review aims at describing the novel concepts and advancements in the fundamental science of VES-based fracturing fluids reported in the last few years, which have not yet been widely industrially implemented, but are significant for prospective future applications. Recent achievements, reviewed in this paper, include the use of oligomeric surfactants, surfactant mixtures, hybrid nanoparticle/VES, or polymer/VES fluids. The advantages and limitations of the different VES fluids are discussed. The fundamental reasons for the different ways of improvement of VES performance for fracturing are described.

## 1. Introduction

Hydraulic fracturing was first introduced in late 1940s as an enhanced oil recovery (EOR) technique [1], and since then, it has become one of the most widely used and effective methods for the intensification of oil and gas inflow to the wells [2,3]. Hydraulic fracturing increases the well debit, e.g., the volume of liquid or gas extracted from the well per unit of time. Currently, it is used both for resuming the exploitation of depleted wells and for bringing new, especially low-permeable, reservoirs into operation. The fracturing fluids can be water-based or non-aqueous [4].

Water-based fracturing fluids consist of a viscoelastic liquid or a gel in which proppant particles (sand or ceramic particles up to a few millimeters in size) are suspended [5,6]. At the first stage of a fracturing operation, the fluid is injected into the wellbore under high pressure and creates fractures in the oil-bearing layer that propagate in the accumulations of oil located far from the well. At the next stage, the pressure is removed, but the fracture remains open and filled with proppant particles with a large number of pores/channels between them. The fracturing fluid remaining between the particles must be broken and cleaned out from the pores. After that, the hydrocarbons can flow to the well through the pores between the proppant particles. The fracturing operation enlarges the area from which the hydrocarbons are produced, allows the extraction of the hydrocarbons from the accumulations located far from the well and, thus, substantially increases the rate of oil extraction.

For a successful fracturing operation, the fluids should meet the following criteria [7] and should:

(1) Form a stable proppant suspension, prevent its sedimentation, and efficiently transport it into the fracture, which is achieved by high viscosity and elasticity;
(2) Be easily pumped into the wellbore with a minimal pressure drop, for which shear thinning behavior is important;
(3) Pass through perforations without degradation of the mechanical properties, for which recovery after high shear is necessary;
(4) Have sufficient temperature resistance, e.g., not degrade viscosity upon heating to reservoir temperatures;
(5) Show sufficient salt tolerance, e.g., dissolve in water in the presence of salts (e.g., sodium, calcium and magnesium chlorides, or sulfates);
(6) Be easily cleaned out from fracture after it is created, for which the fluids should decrease the viscosity and elasticity under the action of an external stimulus (breaker) or upon contact with reservoir hydrocarbons;
(7) Not damage the formation;
(8) Be compatible with the formation;
(9) Have minimal environmental impact.

An absolute majority of the water-based fracturing fluids currently use polymers as thickening agents [8,9]. Both natural and synthetic polymers are employed, and they may be linear (un-cross-linked) or gelled (cross-linked). Among the natural polymers, polysaccharides are used [10], including guar gum and its derivatives (hydroxypropyl guar-HPG and carboxymethyl hydroxypropyl guar-CMHPG); xanthan gum; cellulose derivatives (hydroxyethyl cellulose, carboxymethyl cellulose, and carboxymethyl hydroxyethyl cellulose); etc. Cross-linkers may include various multivalent metal ions (Zr^4+^, Ti^4+^, Cr^3+^, Al^3+^, Fe^3+^, and Fe^2+^) [10] or borate ions for guar-based fluids [11]. Among the synthetic polymers, polyacrylamide and its derivatives [12], including hydrophobically modified polyacrylamide (HM PAAm) [13,14], are mainly employed.

However, though the technological processes for polymer-based fracturing fluids are well-established, they possess some disadvantages which are difficult to overcome. One of the important problems is the destruction and removal of residual liquid from the fracture (clean-up). For polymer-based fluids, specially introduced substances—breakers—are used, which cause the destruction of the network structure of the gel by breaking up the polymer chains [15]. At present, mainly enzyme or oxidative breakers are employed. Enzyme breakers cleave bonds in the main or side chains of the polysaccharides, leading to their shortening. However, the activity of enzymes can be greatly reduced by changing the external conditions; so, their use is limited. Oxidative breakers (persulfates for example) form free radicals, which also cause the destruction of bonds in the polymer backbone. This involves the use of highly concentrated solutions of oxidants, which may not be ecologically and technologically safe. Moreover, the use of all types of breakers that cut the polymer backbone does not allow complete destruction of the network structure and removal of the gel from the proppant pack.

The exploitation of unconventional reservoirs, including low-permeable, high-temperature, and high-salinity wells [16], has stimulated an extensive search for novel fracturing fluids with enhanced properties. The development of clean and cost-effective fracturing fluids is key for the advancement of the hydraulic fracturing technology. Currently, viscoelastic surfactants (VES) [17,18,19] are the main alternative to conventional polymer-based fluids.

Though commercial, VES-based fracturing fluids already exist and are industrially employed, new basic concepts and fundamental scientific advancements have been proposed in the last few years and are the focus of this article. Recent efforts to improve the properties of VES-based fluids to extend their use in hydraulic fracturing are reviewed, with particular attention given to the oligomeric surfactants and mixed (including nanocomposite) systems. These new VES-based fluids have not yet found vast application in the oil industry but are of great potential if their enhanced properties can be combined with a moderate cost and availability of the chemicals used (which, among other issues, implies simpler methods of synthesis of new surfactants, polymers, etc.). The fundamental scientific basis of the different ways to improve the VES performance in fracturing operations is discussed.

## 2. General Concepts of VES-Based Fluids

Since their introduction in 1997 [20,21], polymer-free, surfactant-based fracturing fluids have been attracting constantly increasing attention due to their adequate viscoelastic properties and sand-carrying capacity combined with almost complete clean-up from fracture and low formation damage [22,23,24]. Such fluids contain VES which are able to thicken aqueous solutions due to the formation of long self-assembled aggregates—wormlike micelles (WLMs) [25].

Surfactant molecules are amphiphilic and contain two parts—a polar hydrophilic head and a non-polar hydrophobic tail. Above the critical micelle concentration (CMC), they self-assemble and form aggregates of various shapes—spherical or cylindrical micelles, lamellae, bicontinuous structures, etc. [26]. The cylinders can grow and form very long chains—WLMs—which resemble polymers [27] and can attain several microns in length. Above an overlap concentration (C*), WLMs form an entangled network, imparting high viscosity and viscoelastic properties to water solutions [28]. Zero-shear viscosities up to 5–7 magnitudes higher than the viscosity of water are attained at moderate surfactant concentrations in the range of a few wt %, making VES highly efficient thickeners for fracturing fluids and comparable to polymer-based fluids [29].

WLMs may be formed by ionic or non-ionic surfactants; however, most of the examples of fracturing fluids currently employed contain ionic VES. Normally, WLMs are formed by ionic surfactants in the case of sufficient screening of electrostatic repulsions between similarly charged head groups, which allows them to pack closer than in the spherical micelles [25]. Screening of electrostatic forces may be achieved by the addition of low-molecular-weight salts (such as KCl) [30,31,32], oppositely charged surfactants [33], or hydrotrope ions [34,35,36].

Surfactants containing long (C18–C22) alkyl chains are currently regarded as the best thickening agents for fracturing fluids. Examples of such surfactants include a cationic surfactant with C22 mono-unsaturated tail - erucyl bis-(hydroxyethyl)methylammonium chloride (EHAC) [30] and an anionic surfactant with C18 mono-unsaturated tail – sodium (or potassium) oleate [37].

Another possibility for forming WLMs consists in the use of zwitterionic surfactants, which contain both anionic and cationic groups in the polar head. Such VES are generally more expensive than cationic or anionic species, but they provide stronger thickening effect at lower concentrations. Betaine surfactants with long (C22) tails are among the best thickening agents [29,38,39,40].

Examples of non-ionic surfactants forming WLMs include mixtures of polyoxyethylene alkyl ethers [41], single ultra-long chain (C18–C24) alkyl ethers [42], sugar-based surfactants [43], cholesterol-based surfactants [44], etc.

## 3. Advantages and Limitations of VES-Based Fluids

VES solutions have a number of advantages over polymer fracturing fluids [24]. They arise from the fact that, in contrast to polymer chains, the wormlike surfactant micelles are labile structures formed by the self-assembly of small molecules due to weak non-covalent interactions. Therefore, they are dynamic and are able to break and recombine [45]. This imparts reversible shear-thinning to the VES solutions: their viscosity decreases by several orders of magnitude under shear, which is explained by the alignment and breaking of the WLMs in flow, but completely restores at rest. This property is very important in hydraulic fracturing operations to facilitate the pumping of the fluid into the well.

Another advantage of the self-assembled nature of WLMs consists in their high responsiveness to external factors, e.g., to the presence of hydrocarbons [46,47]. Hydrocarbons are solubilized inside hydrophobic micellar cores, which leads to the change of surfactant molecular packing and the transformation of the WLMs to small (several nm in size) microemulsion droplets [48,49]. This is accompanied by a drastic drop of viscosity to the values close to the viscosity of water and by a complete disappearance of the viscoelastic properties. Due to this, VES solutions are called “clean fluids” as in most cases they require no breakers to be removed from the well after fracture formation. Reservoir hydrocarbons serve as a breaker and completely destroy the WLMs, leading to an almost complete fracture clean-up.

However, the labile structure of the micellar chains also imparts some limitations, which include weaker viscoelastic properties than those of the polymer fluids and poor temperature and salt stability.

Therefore, recent efforts in the research on VES fluids have been directed at solving these problems. The main approaches consist in the use of oligomeric surfactants or of mixtures of several surfactants or the addition of nanoparticles (NPs) or polymers to the VES fluids.

## 4. Oligomeric Surfactants

One of the approaches to increasing the strength of micellar networks for hydraulic fracturing applications is the use of oligomeric surfactants [50]. These surfactants consist of several hydrophobic tails (n) and hydrophilic heads (m) covalently linked to each other [51]. Such molecules exhibit stronger hydrophobic interactions, which enhance micellization, resulting in lower CMC values and smaller amounts of non-aggregated free surfactant, the lower surfactant concentrations necessary to form WLMs, and higher micellar scission energies and more stable micelles.

With regard to the example of cationic surfactant oligomers with quaternary ammonium heads, it was demonstrated that with an increase in the number of units in the oligomer (n), the CMC decreases, and the packing of surfactant molecules in the micelles becomes denser [52]. With an increase in n from 2 to 4, a transition occurs first from the spherical to the cylindrical micelles, which is accompanied by a significant increase in viscosity, and then to the ring-like micelles. Longer alkyl spacer—(CH_2_)_s_—between head groups of quaternary ammonium gemini surfactants also decreases the CMC [53]. An increase in the concentration of dimeric or trimeric surfactants leads to a transition from spherical aggregates to cylindrical and then to long WLMs, as shown by molecular dynamics simulations [54,55].

A promising approach consists in the synthesis of oligomeric surfactants with very long (C18–C22) alkyl tails in order to enhance the hydrophobic interactions. For instance, gemini cationic surfactants EHAB 22:1-s-22:1 (based on two erucyl dimethylammonium bromide moieties linked by an alkyl spacer) with C22 unsaturated tails and different spacers (s = 2, 6) were synthesized, and it was shown that the solutions of gemini EHAB exhibit higher viscosities at lower surfactant concentrations than the monomeric EHAB, which is due to the easier formation of longer WLMs [56]. Addition of a hydrotrope ion (sodium salicylate) to the gemini EHAB 22:1-6-22:1 allows the reaching of the zero-shear viscosity of ca. 30,000 Pa·s.

Recently, a series of dimeric and trimeric VES with long tails have been proposed, aiming at the increased temperature and salt resistance of WLMs [57,58]. The use of a trimeric cationic surfactant with long hydrophobic tails based on erucamidopropyldimethylamine allows the obtaining of WLMs with good thermal stability—due to very strong hydrophobic interactions the micelles do not break down when heated to at least 90 °C, and a stability of the solutions under ultra-high temperatures ranging from 140 to 180 °C is observed [59]. High thermal stability (up to 110–150 °C) was achieved for long-chain (C22) gemini surfactants with various spacers between the polar heads (C25-alkyl(C6)-C25, C25-alkyl(C8)-C25, C25-ester-C25, and C25-amide-C25) [60,61]. The incorporation of a sulfonate group into the spacer of a gemini zwitterionic VES with two unsaturated C22 tails resulted in a significant increase in the salt tolerance as compared to the previously reported cases [62]. Addition of a benzene sulfonate group in the polar head of a gemini zwitterionic surfactant with C22 tails (EDBS) allowed the obtaining of a viscoelastic WLM solution with combined good temperature and salt resistance: it withstood shearing at 170 s^−1^ and 120 °C for 120 min in the 25% standard brine solution [63].

Thus, a variety of gemini and trimeric surfactants with C22 erucyl tails and different chemical structures of spacers between polar groups were proposed (Table 1). However, the variation of the tail length may also be of importance in obtaining an optimized composition for the fracturing fluids. A gemini surfactant with 18:1 oleyl tails was proposed and showed sufficient viscoelastic properties and shear resistance at 80 °C, low proppant settling velocity, and low permeability and conductivity loss rates [64]. However, the behavior of the fluid at higher temperatures (above 80 °C) was not studied. The viscosity of the fluid at 80 °C reached 70.2 mPa·s, and should be lower at higher temperatures, which may be insufficient for high-temperature applications of the fluid.

A promising improvement of gemini surfactants consists in the synthesis of dissymmetric surfactants containing different alkyl tails. For instance, a heterogemini cationic surfactant, YS-18, containing one saturated (18:0) and one unsaturated (18:1) tail, has much better salt tolerance and thermostability than the corresponding symmetrical surfactants. YS-18/KCl fluid viscosity remains stable under a share rate of 170 s^−1^ at 110 °C for 60 min and can easily be broken by reservoir brine and hydrocarbon [65].

Tariq et al. [67,68,69] showed that the use of a solution of cationic gemini surfactants with different spacers resulted in the reduction in the breakdown pressure in unconventional tight sandstones compared to that of the deionized water, which was attributed to the clay stabilization against swelling provided by the surfactant. In addition to the lower breakdown pressure, the use of gemini surfactants allows a reduction in the volume of fluid required to fracture the formation. The coreflooding experiment used to evaluate the formation damage demonstrated that the gemini surfactants do not cause any permeability impairment.

Therefore, the use of oligomeric surfactants with long hydrophobic tails is a promising way of improving the properties of WLMs. Incorporation of a sulfonate group or/and a benzene ring in the spacer increases the temperature and salinity stability of the VES fluids. Cationic gemini surfactants reduce the breakdown pressure and do not cause any permeability impairment. Up to now, mostly dimeric (*n* = 2) and trimeric (*n* = 3) surfactants have been employed due to the difficulties associated with the synthesis of higher oligomers. Development of new oligomeric surfactants with *n* ≥ 4 at a reasonable cost may further advance the fracturing fluid applications of oligomeric surfactants.

## 5. Mixtures

### 5.1. Single-Chain Surfactants

A common method of WLM preparation consists in mixing different surfactants—anionic/cationic [70], zwitterionic/anionic [71,72], anionic/non-ionic [73], cationic/non-ionic [74,75], or two non-ionic surfactants [44]. In many cases, mixing results in the synergistic enhancement of the viscoelastic properties.

Strong synergy is observed for many anionic/cationic surfactant systems, even in the absence of added low-molecular-weight salt, and is a result of two processes: (1) the anionic and cationic polar heads reside in close proximity due to the co-assembly of two surfactants, resulting in a very efficient screening of the electrostatic repulsions at the micellar surface and tighter packing of the surfactant molecules, and (2) the counter ions of both surfactants are released to the solution, giving additional salt, which also weakens the electrostatic repulsions. For instance, in the mixtures of sodium or potassium oleate with different alkyltrimethylammonium or alkylpyridinium surfactants (*n*-octyltrimethylammonium bromide [33], *n*-octylpyridinium chloride [76], 1-dodecylpyridinium chloride [77]), the formation of a viscoelastic network of WLMs with viscosities up to ca. 1000 Pa·s was observed, which was not inherent in the solutions of each surfactant taken separately. Many other viscoelastic mixed systems of anionic/cationic surfactants have been described: cetyltrimethylammonium tosylate/sodium dodecyl benzyl sulfonate [78,79]; hexadecyl- or dodecyltrimethylammonium bromide/N-dodecylglutamic acid salt [70]; cetyltrimethylammonium bromide with sodium laurate [80,81,82]; sodium dodecyl sulfonate [83] or sodium N-alkylmaleimidepimaric carboxylate [84]; and cetylpyridinium chloride/sodium deoxycholate [85] systems. If the association between the anionic and cationic species is rather strong, such a co-assembly may be treated as a formation of a “pseudo-gemini” betaine surfactant. At a non-stoichiometric ratio of anionic and cationic surfactants, the system to some extent resembles mixtures of a betaine and a single-chain ionic surfactant.

A great advantage of mixed anionic/cationic WLMs is a strong rheological synergy, due to which sufficient viscoelastic properties are attained at a reduced concentration of surfactants. However, these systems are usually prepared without additional low-molecular-weight salts and, thus, are salt-sensitive. Positive cases of viscosity and elasticity increase upon the addition of monovalent salts (KCl) have been reported [33]; however, due to the presence of an anionic surfactant, anionic/cationic WLMs may be intolerant to divalent cations (Ca^2+^, Mg^2+^, etc.).

Therefore, other mixed surfactant systems may be advantageous for potential applications, and examples of recently studied systems include mixtures comprising oligomeric surfactants.

### 5.2. Oligomeric and Single-Chain Surfactants

One of the approaches for obtaining long WLMs consists in the mixing of oligomeric surfactants with single-chain species [86,87]. A synergistic effect has been observed for some short-chain (C12) cationic oligomeric surfactants mixed with anionic single-chain species. For instance, the addition of small amounts (5–20 mol%) of single-chain anionic surfactants (sodium dodecyl sulfate, sodium dodecyl benzene sulfonate, and sodium laurate) to a cationic gemini surfactant 12-3(OH)-12 (2-hydroxyl-propanediyl-α,ω-bis (dimethyldodecylammonium bromide) induces an increase in viscosity by 2 orders of magnitude, up to 60–120 Pa·s, and an enhancement of the viscoelastic properties of the micellar network [88]. In the mixtures of a trimeric cationic surfactant DDTPA (penta sodium N,N′,N′′-dodecyl diethylene triamine pentaacetic acid) and sodium dodecyl sulfate in the presence of KCl, a network of WLMs is formed, which imparts high zero-shear viscosity (~400 Pa·s) and viscoelastic behavior [86].

### 5.3. Multiple Oligomeric Surfactants

Very recently, the mixing of two different long-chain (C22) sulfobetaine zwitterionic gemini surfactants, EDABS (based on erucyl dimethyl amidopropyl benzenesulfonic acid) and EDAES (based on erucyl dimethyl amidopropylethanesulfonic acid), which differ only slightly by the presence of a benzene ring in the polar head, was proposed to obtain a combination of viscoelasticity, good shear, and temperature and salt tolerance [89].

### 5.4. “Pseudo-Oligomeric” Surfactants

As stated above (Section 5.1), a strong association of anionic and cationic single-chain surfactants may be treated as a formation of a “pseudo-gemini” betaine surfactant species comprising anionic and cationic polar groups and two tails from both surfactants. However, such associations are transient and usually sensitive to salts.

A recently proposed interesting approach consists in the non-covalent self-assembly of “ordinary” single-chain cationic surfactant cetyltrimethylammonium bromide (CTAB) into a pseudo-gemini species by an interaction with di- or tricarboxylic acids (citric or maleic acid) [90]. The fluid possesses high viscosity (1000 Pa·s), strong viscoelasticity, and temperature and shear resistance. Such an approach eliminates the complex and expensive synthesis of oligomeric surfactants as cheap surfactants and acids may be used.

Therefore, the mixing of surfactants is an efficient way of obtaining viscoelastic networks of long WLMs with enhanced rheological performance. Promising approaches consist in using mixtures of single-chain and oligomeric surfactants or in the formation of “pseudo-oligomeric” surfactants.

## 6. Nanoparticle-Enhanced VES Fluids

The rheological properties of VES can be significantly enhanced by added nanoparticles (NPs), leading to the extended stability of the VES fluids in withstanding high shear rate and temperature during the fracturing process, minimization of fluid loss, and improvement of sand-carrying ability [17,91].

**Figure 1:**
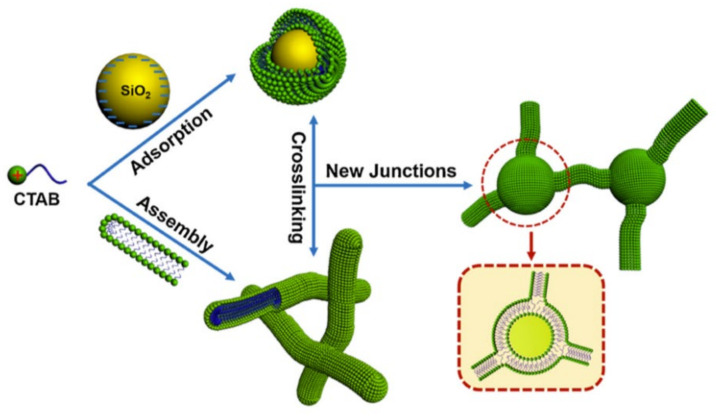
Schematic representation of the mechanism of nanoparticle-enhanced VES fracturing system on an example of a cationic surfactant cetyltrimethylammonium bromide (CTAB) WLMs interacting with anionic silica NPs. Reproduced from Zhang Y., Dai C., Qian Y., Fan X., Jiang J., Wu Y., Wu X., Huang Y., and Zhao M. Rheological properties and formation of dynamic filtration damage evaluation of a novel nanoparticle-enhanced VES fracturing system constructed with wormlike micelles. *Colloids and Surfaces A: Physicochemical and Engineering Aspects 553*, 244–252, Copyright (2018), with permission from Elsevier.

The enhancement of the rheological properties results from the binding of micellar end-caps to the surface of the NPs, leading to elongation or cross-linking of the micelles by the NPs (Figure 1) [92]. WLMs can interact with a large variety of NPs regardless of their size, shape, and surface functionality [93]. When the NPs are oppositely charged with respect to the surfactant ions, the electrostatic interactions govern the adsorption, and the surfactant is attached to the NPs via its charged head-groups, leaving the hydrophobic tails outside. To exclude the contact of these hydrophobes with water, a second surfactant layer is attached in such a way that the hydrophobic tails face the tails of the first layer, leaving the hydrophilic heads in contact with the water. Thus, in the case of NPs oppositely charged with respect to the surfactant ions, the surfactant double layers are formed. The formation of the double layer results in the charge reversal of NPs, as was demonstrated by the ζ-potential measurements [94]. When the NPs are hydrophobic, the hydrophobic interactions govern the adsorption [95], and the surfactant is attached to the NPs by its hydrophobic tails, forming a monolayer with the polar groups exposed to water. The surfactant can adsorb on the NPs even if they are similarly charged, due to the hydrophobic interactions; in this case, hemimicelles are formed [96].

The NPs covered by surfactant can further interact with the WLMs. According to the theoretical studies [97], the interaction proceeds via the fusion of the semi-spherical end-caps of the WLMs with the surfactant aggregates adsorbed on the surface of the NP (Figure 1). The end-caps are mainly involved in the interaction because they represent the energetically unfavorable parts of the micelles where the head-groups are located rather far from each other, allowing penetration of some water molecules into the micelle. Direct evidence of the linking between the WLMs and the NPs was provided by cryo-transmission [96,98] and freeze-fracture transmission electron microscopy [99].

**Figure 2:**
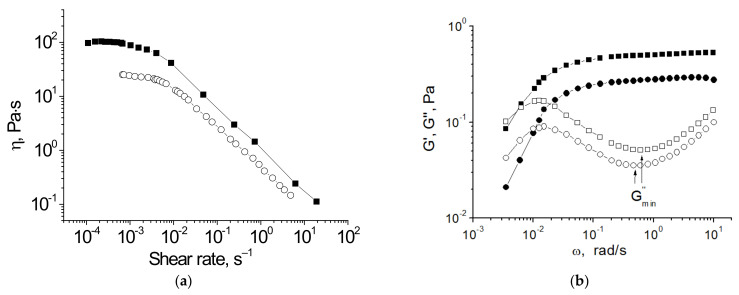
Viscosity as a function of shear rate (**a**) and frequency dependences of storage modulus G’ (filled symbols) and loss modulus G” (open symbols) (**b**) for 0.6 wt % erucyl bis-(hydroxyethyl) methylammonium chloride solutions with 1.5 wt % 300 nm magnetite particles (squares) and without nanoparticles (circles) at 20 °C. Solvent: 1.5 wt % KCl in water, pH 11. Reprinted with permission from Pletneva V.A., Molchanov V.S., Philippova O.E. *Langmuir* 2015, *31*(1), 110–119. Copyright (2015) American Chemical Society.

The NPs can increase the zero-shear viscosity η_0_, relaxation time τ_rel_, and the plateau modulus G_0_ of VES fluids (Figure 2) [94,96,98,99,100,101,102,103,104,105,106,107,108,109,110,111]. The most pronounced is the effect of NPs on the viscosity, which can augment by up to 3 orders of magnitude [94,96,98,99,100,104,105,106,107,108,110,111]. It can be attributed to the hindered reptation of WLMs as their motion slows down in the vicinity of NPs. The largest enhancement of viscosity is observed for VES fluids in the vicinity of the overlap concentration C* of WLMs. In these conditions, the micelles are rather short, and therefore, in the system, there is a large quantity of end-caps. When the NPs are added, they interact with the micellar end-caps, thereby building a network. The increase in viscosity by 3 orders of magnitude, leading to the transition of a VES solution into an elastic gel, was observed for anionic WLMs of fatty acid methyl ester sodium sulfonate (MES) and 20–40 nm barium titanate NPs [99]. In semi-dilute solutions of WLMs, the enhancement of the viscosity by added NPs usually does not exceed 1.5 orders of magnitude [94,104,105,106,107]. It was shown that for ionic VES the increase in viscosity by NPs is more pronounced at lower salt concentrations because the micelles become shorter and the quantity of end-caps in the system increases. For instance, for a 0.6 wt % solution of C22-tailed cationic surfactant erucyl bis-(hydroxyethyl) methylammonium chloride, the enhancement of viscosity by 5 wt % 300 nm magnetite particles is 12.3-fold and 7-fold in 1 and 1.5 wt % KCl, respectively [104]. 

In some papers [94,105,107,111], it was observed that upon the gradual increase in the amount of added NPs (at a constant concentration of surfactant) the zero-shear viscosity first increases, reaches a maximum, and then starts to decline. For instance, in VES fluid composed of a C22-tailed cationic surfactant N-erucamidopropyl-N,N-dimethyl-N-allylammonium bromide (EDAA) and negatively charged SiO_2_ particles, the maximum value of viscosity was observed at as low an amount of NPs as 0.01 wt %, and the further increase in the NP concentration resulted in the decrease in viscosity, which became even lower than the viscosity of the initial VES solution [94]. In VES fluid consisting of a 3 wt % sodium oleoyl methyl taurate (SOMT), 6 wt % NaCl, and 22 nm SiO_2_ particles, the maximum value of viscosity was observed at 0.9 wt % NPs [105]. The drop in viscosity was attributed to the increased electrostatic repulsion between the WLMs and the NPs covered by the same surfactant [94] or to interparticle aggregation leading to the disruption of the network structure [107]. One cannot exclude [93] the fact that the adsorption of the surfactant molecules on the surface of the added NPs can reduce the amount of surfactant involved in the formation of WLMs, which should also significantly contribute to the lowering of the viscosity. In these systems, the relaxation time τ_rel_ passes through the maximum simultaneously with viscosity [105]. The τ_rel_ value in the network of entangled WLMs is determined by the breaking time of the micelles τ_br_ and the reptation time τ_rep_ [29]. It was shown that NPs do not appreciably affect the breaking time of the micelles τ_br_ [105] or even decline it [104]. Therefore, the increase of τ_rel_ is caused by the increase of reptation time τ_rep_ provided by the hindered reptation of WLMs in the presence of NPs.

As to the plateau modulus G_0_, it either monotonically increases with the increasing amount of added NPs [96] or first increases and then reaches a constant value [104,105]. The increase of G_0_ was attributed to the cross-linking of WLMs by NPs adsorbing the micellar end-caps. When all the micellar end-caps available in the system are linked to NPs, G_0_ reaches a constant value. Further addition of particles leads only to a redistribution of micellar end-caps between the NPs, which does not produce new elastically active sub-chains [104]. The increase of G_0_ induced by NPs is usually rather small: less than two-fold [96,99,104,105]. This behavior can be explained as follows [96]. If before the addition of NPs each micelle had n elastically active sub-chains due to entanglements with other micelles, the binding of its two end-caps to NPs would give two more elastically active subchains, thereby providing a ca. (n + 2)/n-fold increase of the plateau modulus. The lower the number of initial entanglements between the WLMs, the higher the input of NPs in G_0_ value. Thus, being taken in a proper amount, the NPs can increase the viscosity η_0_, the relaxation time τ_rel_, and, to a lesser extent, the plateau modulus G_0_ of the VES fluids due to the attachment of the energetically unfavorable end-caps of the micellar chains.

NPs can also enhance temperature and shear resistance, which is quite important for fracturing fluids. These parameters can directly influence the fracture-making and sand-carrying capabilities of the fluid [112]. Wu and co-workers [94] examined the temperature and shear resistance of VES fluids containing 1 wt % cationic surfactant EDAA and 0.01 wt % negatively charged SiO_2_. The experiments were performed at a shear rate of 170 s^−1^ and at 70 °C. It was shown that for 2 h the fluid with the NPs keeps a rather high shear viscosity of 33 mPa·s, whereas without NPs the shear viscosity was only 24 mPa·s. Note that the increase in viscosity in the presence of NPs was due to higher longest relaxation time τ_rel_ (0.15 s) exceeding that of the VES without NPs (0.054 s). The static particle settling test showed that the EDAA/silica system has a lower settlement rate (0.0021 cm/s) as compared to the pure EDAA solution (0.012 cm/s) and other fracturing fluids, such as guar gum (0.72 cm/s), thereby demonstrating a good proppant-carrying performance.

Zhang et al. [110] studied the effect of NPs on the shear restoration of VES fracturing fluid, demonstrating its ability to recover viscoelasticity after high-speed shearing during pumping. For the fluid composed of 30 mM of cetyltrimethylammonium bromide and 40 mM of sodium salicylate NaSal, it was shown that the addition of 0.1 wt % 140 nm silica NPs increases the shear recovery rate from 83.4% to 92.3%. Therefore, NPs favor faster reconstruction of micellar networks at low shear rates.

Huang and Crews found that NPs added to VES fracturing fluid produce a pseudo-filter cake due to the aggregation with the micelles, thereby significantly reducing the rate of fluid loss and improving fluid efficiency [102]. At the same time, the same fluid without NPs was non-wall-building and had very high fluid leak-off over time [102]. Similar filter cake formation in the presence of NPs was observed by Zhang et al. [110]. It is important to note that the filter cake thus formed can be easily and completely removed at the flow of oil, leaving very little residue or production damage [102].

For enhancement of the performance of VES fluids, different types of NPs were used, including silica SiO_2_ [94,96,100,105,110,111], barium titanate BaTiO_3_ [99], magnetite Fe_3_O_4_ [104,109,113], MnO [114], ZnO [103,114], TiO_2_ [115], and carbon nanotubes [108]. It was shown that among these NPs, long nanotubes induce a smaller increase in viscosity than spherical NPs [108]. This can be due to the sliding of the WLM-nanotube junctions along the nanotube, thus releasing the stress and reducing the viscosity.

In all the discussed papers above, the NPs were added to the WLMs of the surfactants. At the same time, the addition of NPs to the VES systems with lamellar structure was also examined [87]. For instance, Baruah et al. studied VES-based fracturing fluids composed of zwitterionic (cocamidopropyl betaine) and anionic (sodium oleate) surfactant mixtures, iso-amyl alcohol as a cosurfactant, clove oil, and water. It was shown that the addition of 0.1% SiO_2_ and 0.1% NaOH leads to the increase in apparent viscosity by 5–45%, depending on the temperature and surfactants ratio. The authors explained this behavior by the penetration of the SiO_2_ nanoparticles within the lamellae structure of the VES fluids, which helps the development of a 3D network and enhances the interconnections between the mixed surfactant monomers constituting the lamellar liquid crystal phase. The NPs act as a swelling agent that increases the thickness of the water layers in the lamellar structure, making it more stable; in its turn, the lamellar structure stabilizes the dispersion of the NPs. It is interesting that the system was highly pressure-sensitive. At atmospheric pressure, the fluid was unable to maintain an apparent viscosity higher than 90 mPa·s for more than 3 min. However, under higher pressures (2068, 4137, and 6205 kPa), it kept an apparent viscosity higher than 600 mPa·s for 120 min at 103 °C and 100 s^−1^ (this shear rate is associated with the shear which the fluid should experience during pumping in the well), which is suitable for conducing the fracturing job and to suspend proppants.

Thus, the addition of NPs seems to be an effective way to improve various properties of VES-based fracturing fluids.

## 7. Hybrid Polymer–VES Fluids

### 7.1. Interaction of Polymers with VES

One of the new directions in the research on hydraulic fracturing fluids is aimed at combining the advantages of polymer- and surfactant-based fluids, which may be achieved by creating mixed (or “hybrid”) gels of polymer chains and WLMs. A key factor determining the structure and properties of such fluids is the interaction of the polymer and surfactant. The tuning of this interaction allows the obtaining of homogeneous mixtures and the preserving of the structure of micellar chains.

The addition of polymers may either destroy or preserve WLMs, depending on the hydrophobicity of the polymer. For instance, weakly hydrophobic but water-soluble polymers, such as poly (vinyl methyl ether) or poly (propylene oxide), break WLMs [116,117,118]. This happens due to the “wrapping” of the polymer chains around the micelle, which is favorable because it reduces the contact of both the micellar and the polymer hydrophobic parts with water. As a result, the WLMs are transformed into spherical [119], ellipsoidal [116], or disc-like aggregates [120].

Some polymer molecules containing hydrophobic groups along the backbone, such as partially sulfonated polystyrene [121], poly(methyl methacrylate-co-sodium styrene sulfonate) copolymer [122], or sodium poly(p-vinylbezoate) [123], can embed into WLMs without their disruption, forming stronger “hybrid” micelles armed by polymer: a more hydrophobic polymer backbone is solubilized in the micellar core, while charged polymer units reside closer to the oppositely charged surfactant head groups, screening electrostatic interactions at the micellar surface. The formation of “hybrid” polymer-surfactant cylindrical micelles was also observed for water-insoluble polymer poly(4-vinylpyridine) [124,125,126].

However, the most practically important cases comprise water-soluble polymers which do not break WLMs. Two types of such systems have been reported: hydrophobically modified (HM) polymers or fully hydrophilic polymers which do not interact with the micelles.

### 7.2. HM-Polymers/VES

**Figure 3:**
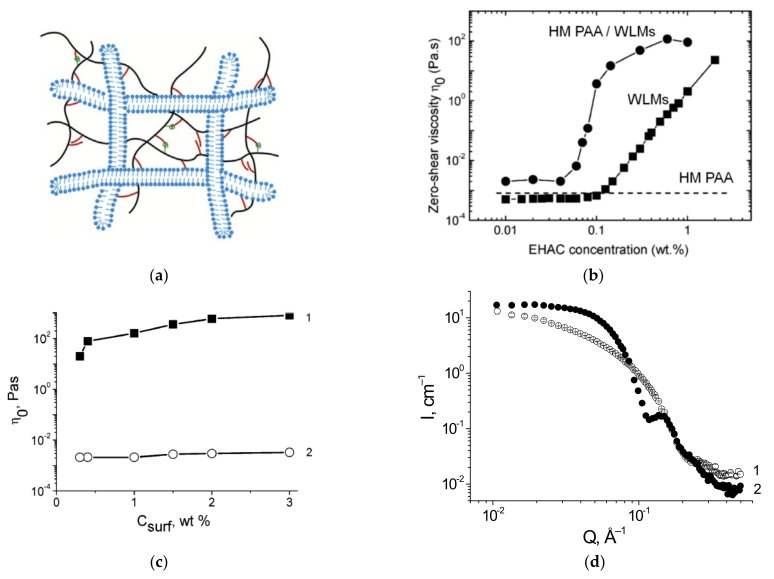
(**a**) Schematic representation of a common network of wormlike surfactant micelles (WLMs) and hydrophobically (HM) modified polymer; Reproduced from Pu, W.; Du, D.; and Liu, R. Preparation and evaluation of supramolecular fracturing fluid of hydrophobically associative polymer and viscoelastic surfactant. *J. Petrol. Sci. Eng. 167*, 568–576, Copyright (2018), with permission from Elsevier; (**b**) zero-shear viscosity as a function of cationic surfactant (EHAC) concentration for the mixtures of WLMs and 0.5 wt % HM PAAm and components (HM PAAm and WLMs) taken separately; solvent: 3 wt % KCl in water, temperature: 60 °C; reprinted and adapted with permission from Shashkina, J.A.; Philippova, O.E.; Zaroslov, Y.D.; Khokhlov, A.R.; Pryakhina, T.A.; and Blagodatskikh, I. Rheology of viscoelastic solutions of cationic surfactant. Effect of added associating polymer. *Langmuir 21*(4), 1524–1530, Copyright (2005) American Chemical Society; (**c**,**d**) zero-shear viscosity as a function of anionic surfactant (potassium oleate) concentration (**c**) and small-angle neutron scattering curves (**d**) for the mixtures of WLMs and 0.5 wt % HM PAA before (1) and after (2) addition of n-dodecane; solvent: 6 wt % KCl in water, temperature: 20 °C; reprinted with permission from Molchanov, V.S.; Philippova, O.E.; Khokhlov, A.R.; Kovalev, Y.A.; and Kuklin, A.I. Self-assembled networks highly responsive to hydrocarbons. *Langmuir 23*(1), 105–111, Copyright (2007) American Chemical Society.

HM-polymers usually have a main hydrophilic water-soluble backbone and some amount of grafted hydrophobic water-insoluble groups. Such polymers mainly interact with WLMs via their hydrophobic moieties, which can embed into the micelles without their disruption. In this case, common networks of HM-polymer and WLMs can be formed (Figure 3a), and polymer hydrophobic groups serve as “physical” cross-links between the macromolecules and the WLMs. This results in the increase in viscosity and the synergistic enhancement of the viscoelastic properties and temperature stability.

#### 7.2.1. HM PAAm/Surfactants

In most of the published works, synthetic polymers, such as HM PAAm [127,128,129,130,131], hydrophobically modified polyacrylic acid (HM PAA) [132,133,134,135], their copolymers [136,137,138], copolymers of PAAm, and other monomers [139,140,141] are used. Such polymers are usually synthesized by radical co-polymerization of a hydrophilic (acrylamide/acrylic acid) and hydrophobic (e.g., alkylacrylates) monomers in a common solvent [142]. Otherwise, micellar co-polymerization is employed [143].

In a pioneering work [128], HM PAAm with *n*-dodecyl side chains was mixed with C22-tailed cationic surfactant EHAC, and a huge synergistic increase in viscosity by 4 orders of magnitude was observed, and a rise of viscosity began at lower surfactant concentrations than in the absence of polymer (Figure 3b). This behavior is observed at the rather low HM PAAm concentration of 0.5 wt %, close to C*, at which polymer solutions without surfactant have low viscosities close to water. Such a strong synergistic effect was explained by the formation of a common polymer-micellar network with hydrophobic polymer groups serving as junctions to WLMs.

It is shown that the presence of hydrophobic groups is important for the phase compatibility of PAAm and WLMs [128], and the one-phase region widens with an increase in the length and number of grafted alkyl chains. It confirms that polymer hydrophobic groups are responsible for the interaction with WLMs. The distribution of hydrophobic groups along the polymer chain affects the viscoelastic properties of the mixtures [128]: at a fixed total content of hydrophobic groups, statistical HM PAAm induces a stronger increase in the rheological parameters (viscosity, elastic modulus, and relaxation time) than HM PAAm with a higher degree of blockiness, which is due to the formation of more cross-links between the statistical polymer and WLMs. It should be noted that the characteristic lifetime of such cross-links is mainly determined by the WLM breaking time and not by the time when the polymer hydrophobic group resides inside the micelle.

Thus, the main practically important effect of mixing HM PAAm/HM PAA and WLMs consists in the synergistic increase in the viscoelastic properties. Many works showing a similar effect for the mixtures of HM PAAm or its copolymers and anionic [136,137], zwitterionic [138,140,141], zwitterionic and anionic [139], gemini cationic [144], and trimeric zwitterionic [145] surfactants have been published.

SANS data show that the WLM local structure is preserved in the presence of the HM polymer: the SANS curve of mixed WLM/HM PAAm systems is described by a cylinder model with a radius equal to the radius of WLMs in the absence of HM PAAm [136]. An increase in viscoelasticity was observed both for the C8-C12 polymer hydrophobic groups and for a specific case of HM PAAm modified by ultra-long C22 alkyl groups [135].

The second important effect of mixing HM PAAm and WLMs is the increased temperature stability [128,144]. Upon heating, the viscosity of the polymer/VES mixtures decreases to a lesser extent than that of a VES solution in the absence of polymer. This is explained by the fact that, in contrast to the micellar chains, macromolecules do not break upon heating and contribute to the heating resistance of the fluids.

The third advantage of HM PAAm/WLM fluids is their high responsiveness to hydrocarbons [128,136,144]. Upon contact with aliphatic oils, the fluids completely lose viscoelastic properties, and their viscosity drops by several orders of magnitude, reaching the viscosity of water (Figure 3c) [128,136]. The responsiveness of mixed polymer/WLM fluids is due to the hydrocarbon-induced breaking of the WLM sub-chains into spherical microemulsion droplets (which is proven by SANS, Figure 3d), leading to the disruption of the common network. This effect is analogous to the case of WLMs without polymers. In this regard, mixed polymer/WLM solutions are clean fracturing fluids which do not require additional breakers for their removal from the fracture.

An interesting particular case reported in the literature consists in the replacement of the polymer alkyl hydrophobic moieties by different groups. In the work [145], β-cyclodextrin-functionalized HM PAAm was mixed with a trimeric zwitterionic surfactant (DTPAN), and a pronounced increase in viscosity was observed as compared to the components (polymer or VES) taken separately. The fluid was characterized by a favorable temperature tolerance and shearing resistance and an affordable proppant-carrying capacity and was completely broken by ammonium persulfate.

CO_2_-responsive common networks of HM PAAm and WLMs of sodium dodecyl sulfate (SDS)–N,N,N′,N′-tetramethyl-1,3-propanediamine (TMPDA) were reported [146]. The responsiveness of the common networks is explained by the transition of the spherical to the wormlike micelles of SDS–TMPDA mixtures, which is accompanied by a 400-fold increase in viscosity. Sol-gel transition is reversible for at least five cycles of bubbling and removing of CO_2_.

Therefore, HM PAAm/VES fluids combine the advantages of both components: increased viscoelasticity and temperature stability due to the polymer component, and responsiveness to hydrocarbons inherent in surfactant WLMs.

#### 7.2.2. Other Synthetic HM Polymers/Surfactants

The formation of the common networks with surfactant WLMs was also observed for other synthetic HM polymers. For instance, water-soluble telechelic poly (ethylene glycol) polymers end-modified by hydrophobic fragments have been reported to form common networks with cetyltrimethylammonium toluene sulfonate [147] or cetylpyridinium chloride in the presence of sodium salicylate [148,149,150]. It is shown that the hydrophobic end groups of the polymer embed into WLMs, while its main hydrophilic part resides in water and links two micelles together. As a result, the mixtures show viscoelastic behavior which is not observed for the components taken separately [147].

#### 7.2.3. Natural HM Polymers/Surfactants

The common networks of WLMs and natural HM polymers are much less described than the synthetic macromolecules, though natural polymers are preferable for hydraulic fracturing applications due to their better biodegradability, lower environmental impact and lack of need for polymerization processes.

Hydroxypropyl guar, modified by long C22 alkyl groups (HM HPG), was reported to form common viscoelastic networks with cationic surfactant EHAC in the presence of KCl [151], which resulted in a significant synergistic effect in a wide range of concentrations and at elevated temperatures of up to 60 °C. It is important to note that a rather low degree of hydrophobic substitution (10 hydrophobic groups per macromolecule) was enough to achieve a pronounced synergy in rheological properties.

A similar synergistic effect on viscoelastic properties was observed upon the bridging of cationic cetyltrimethylammonium tosylate WLMs with HM chitosan with C12 alkyl side chains [152], or of cetyltrimethylammonium *p*-toluenesulfonate WLMs with HM hydroxyethyl cellulose [153], and, in the latter case, the HM polymer had a much stronger effect on the solution viscosity than a similar unmodified hydroxyethyl cellulose, proving the incorporation of hydrophobic side groups of macromolecules into WLMs.

An absolute majority of WLM/HM polymer (either synthetic or natural) common networks employed alkyl hydrophobic groups attached to polymer. An interesting variation of the hydrophobic modification approach was proposed [154], in which cholesterol-modified gellan gum was used to strengthen a network of cylindrical micelles formed by a non-ionic surfactant polyoxyethylene cholesteryl ester (ChEO_10_), with the hydrophobic tail of the same chemical structure as the polymer hydrophobic groups. At the same time, cholesterol-modified short-chained dextran was shown to break longer WLMs of ChEO_10_ into shorter ones, leading to a more liquid-like behavior [155].

Therefore, the enhancement of the viscoelastic properties of the WLM networks by HM-polymers is a general effect observed for both the synthetic and the natural polymers bearing alkyl groups of different length (C8–C22) or hydrophobic groups of other chemical natures. This effect is due to the bridging of WLMs by polymer chains and the formation of a common polymer/micellar network. The practical application of this approach may be greatly advanced by the easier and cheaper synthesis methods of HM-polymers.

#### 7.2.4. HM Polymers/Surfactants/Nanoparticles

A ternary viscoelastic fracturing fluid containing HM HPG with C14 alkyl side chains, cationic gemini surfactant 12:0-3-OH-12:0 and 7–40 nm hydrophobic silica NPs with improved characteristics was described [156]. The viscosity of the fluid was increased by the addition of NPs from 37 mPa·s to 154 mPa·s at 80 °C and 170 s^−1^; the presence of the surfactant and polymer improved the interfacial properties (oil–water interfacial tension, hydrophilicity of the model core slices aged in the fluid filtrate); the fluid application showed a good oil permeability loss rate (9.4%) and a fracture conductivity retainment rate (95%); and a significant increase in oil production was obtained in the oilfield test. This new approach combines the effect of NPs, which induce elongation of the WLMs and the synergistic effect of HM polymer and the WLMs and shows that a combined synergy may be achieved by using a multi-component fluid.

### 7.3. Hydrophilic Polymers/VES

A recently described novel approach for the development of viscoelastic polymer/surfactant fluids consists in the use of fully hydrophilic polymers which do not interact with WLMs and do not destroy them.

**Figure 4:**
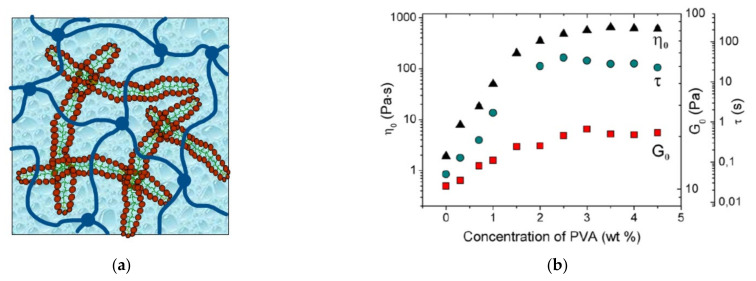
(**a**) Schematic representation of a common network of wormlike surfactant micelles (WLMs) and hydrophilic polymer, which may be un-cross-linked or cross-linked; (**b**) zero-shear viscosity η_0_ (triangles), terminal relaxation time τ (circles), and plateau modulus G_0_ (squares) as a function of concentration of added poly(vinyl alcohol) PVA in 3.3 wt % potassium oleate/C8TAB aqueous solutions at 20 °C, molar ratio [potassium oleate]/[C8TAB] = 2.5; reprinted with permission from Shibaev, A.V.; Abrashitova, K.A.; Kuklin, A.I.; Orekhov, A.S.; Vasiliev, A.L.; Iliopoulos, I.; and Philippova, O.E. Viscoelastic synergy and microstructure formation in aqueous mixtures of nonionic hydrophilic polymer and charged wormlike surfactant micelles. *Macromolecules 50*(1), 339–348, Copyright (2017) American Chemical Society.

One of the first reported cases is the mixing of potassium oleate WLMs with a similarly charged polyelectrolyte-sodium polystyrenesulfonate (PSS) [37]. However, in this work, no increase in viscosity or elasticity caused by the polymer was reported. The WLMs of a cationic surfactant EHAC are preserved in the presence of an oppositely charged natural polyelectrolyte–xanthan [157]. One-phase homogeneous mixtures are obtained when a significant amount (4–4.75 wt %) of low-molecular-weight salt KCl is added, which screens the electrostatic attraction between the components and prevents the precipitation of the polymer/surfactant complex. A synergistic increase of the plateau storage modulus is seen for the mixture, which is explained by the formation of a common semi-interpenetrating network of polymer and micellar chains with entanglements between the components (Figure 4a).

Synergy in viscoelastic properties has been observed for the mixtures of a synthetic polymer poly(vinyl alcohol) and mixed anionic/cationic WLMs [158,159]. A nearly 3-magnitude increase in viscosity is observed, which is accompanied by a significant enhancement of the elastic modulus and relaxation time (Figure 4b) and is explained by two factors: (1) the formation of entanglements between the polymer and micellar chains, (2) the microphase separation with the formation of polymer-rich and micellar-rich microdomains, which arises from weak repulsive interaction between the polymer and micelles. Microphase separation results in the concentration of both components in their domains, leading to the augmentation of the rheological parameters. Later, the same effect was observed for a natural polymer HPG mixed with anionic/cationic WLMs [160]. The use of a weakly repulsive polymer and WLMs, which form one-phase but microscopically segregated solutions, allows the significant reduction in the concentrations of the components in the fluid in order to obtain viscosity and elasticity sufficient for fracturing operations.

A very recently reported novel approach consists in using the hydrophilic polymer/WLM system and cross-linking the hydrophilic polymer chains into their own network (for instance, by borate ions), which leads to the formation of a double dynamic network, consisting of an entangled network of WLMs interpenetrating with a polymer network with labile cross-links [161]. Such polymer/WLM systems are characterized by significantly enhanced rheological properties as compared to their components taken separately—their zero-shear viscosity and plateau storage modulus are increased by factors of 3400 and 27, respectively. Due to the dynamic nature of the bonds in both networks, they recover mechanical properties after strong shear and may be responsive to different factors.

It was proposed to mix the WLM network and cellulose nanofibrils (CNF). Sodium oleate/KCl/CNF fluid was shown to have higher zero-shear viscosity than a simple oleate/KCl fluid, and was able to form a dense filter cake, which reduced filtration of the fluid into the core [162]. A pseudo-interpenetrating network of sodium oleate/KCl WLMs and a CNF surface modified by carboxyl, sulfonic, or hydrophobic groups was prepared and was characterized by higher viscosity than for pure WLMs (at moderate CNF content which does not break the micellar network) and by improved sand-carrying capacity [163].

Thus, the interaction of polymers with WLMs may result in the disruption of the micellar chains, the obtaining of hybrid cylindrical micelles with embedded polymer, or the formation of the common polymer-micellar networks. Among these various structures, common networks of HM polymers and WLMs show the best rheological characteristics (high viscosity and elastic modulus exceeding the values for individual components by several orders of magnitude) and temperature stability combined with a pronounced responsiveness to hydrocarbons. The best properties of mixed fluids are observed when HM polymer contains rather long (C12-C22) alkyl tails or other bulky hydrophobic groups (for instance, β-cycodextrin, or cholesterol) providing a rather strong hydrophobic interaction with WLMs. The synergistic effect of mixing HM polymers and surfactants has already been observed at a rather low amount of hydrophobic substituents in the polymer chain, which may be only a few per macromolecule. However, the application of HM polymer/VES fluids in hydraulic fracturing is limited by a rather high cost and the limited availability of HM polymers. An alternative approach may consist in the use of fully hydrophilic polymers weakly interacting with the micelles.

## 8. Conclusions and Outlook

In this review, several new approaches are described for improving the properties of VES-based fluids, aiming at their application as a cost-effective alternative to polymer-based hydraulic fracturing fluids.

Gemini and oligomeric surfactants show enhanced rheological properties and temperature resistance compared to their single-chain counterparts. Promising ways to obtain better performance of such fluids consist in the use of longer alkyl tails and higher oligomer numbers (*n* ≥ 4), providing stronger hydrophobic interactions within WLMs. Other possibilities include the variation of the polar head chemical substituents, or the use of dissymmetric surfactants, combining alkyl tails of different length within one molecule. A significant advancement in this field would consist in the development of easier synthetic methods, leading to a reduction in the oligomeric VES cost.

A rather simple way for optimizing the properties of VES fluids is the use of surfactant mixtures. One of the prospective routes consists in mixing gemini or oligomeric surfactants with single-chain surfactants or together, which allows the reduction in the concentration of a more expensive oligomeric component while exploiting the synergy between different surfactant species. New interesting solutions may include obtaining “pseudo-gemini” surfactants by self-assembly of multiple cheaper components.

Addition of NPs is an effective and rather inexpensive way for improving the performance of VES fracturing fluids, including the rheological properties (viscosity, plateau shear modulus, and relaxation time), the temperature and shear resistance, and the proppant-carrying capacity. An attractive pathway lies in combining the addition of NPs with oligomeric surfactants, polymer/surfactant mixtures, etc.

The development of hybrid polymer/VES fluids is a widely and fundamentally investigated, but practically unexploited, field. Such fluids comprise mixtures of VES with HM or hydrophilic polymers. Systems comprising synthetic HM PAAm and VES are the most widely studied in the literature; at the same time, mixtures of HM natural polymers with VES are much less investigated. New developments in this field may be related to the cheaper and easier ways of the synthesis of HM polymers, including the use of hydrophobic groups other than alkyl chains, as well as the search for high-performance mixtures of VES with hydrophilic polymers.

Novel approaches in the studies of VES-based fluids offer promising ways for the improvement and optimization of their properties relevant to oil field applications and for the widening of their practical use in hydraulic fracturing technology.

## Figures and Tables

**Table 1:** Reported gemini and trimeric surfactants and their characteristics for hydraulic fracturing applications. Reproduced with adaption from Zhang, Y.; Mao, J.; Zhao, J.; Zhang, W.; Liao, Z.; Xu, T.; Du, A.; Zhang, Z.; Yang, X.; and Ni, Y. Preparation of a novel sulfonic gemini zwitterionic, viscoelastic surfactant with superior heat and salt resistance using a rigid–soft combined strategy, *J. Mol. Liq.* *318*, 114057, Copyright (2020), with permission from Elsevier.

Chemical Structure	Name	Advantages	Limitations	Refs.
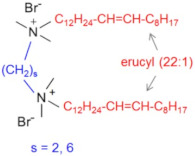	EHAB 22:1-s-22:1	Best characteristics are observed for s = 6: highly viscoelastic or gel-like solutions at high temperatures in the presence of salt (viscosity in the presence of NaSal at 110 °C is greater than 107 mPa·s)	no data	[56]
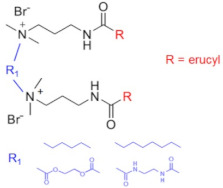	C25-R1-C25	High temperature resistance (up to 110–150 °C)	sensitivity to high salinity	[60,61]
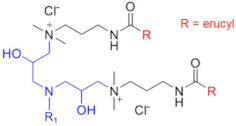	VES-M (R_1_ = methyl) VES-E (R_1_ = ethyl) VES-P (R_1_ = *n*-propyl)	Best characteristics are observed for VES-M with smallest alkyl group in the spacer: - stability up to 139 °C; - no proppant settling for 180 min at ambient temperature; - complete gel breaking by kerosene	sensitivity to high salinity	[57]
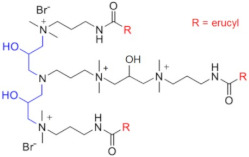	VES-T	- excellent thermal stabilities under ultra-high temperatures ranging from 140 to 180 °C; - gel broken by standard brines within 2 h	no data	[59]
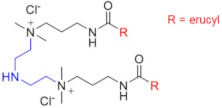	JS-N-JS	- temperature and shear resistance; - gel breaking into a water-like fluid by hydrocarbons; - low damage to the tight sand reservoirs	sensitivity to high salinity	[58]
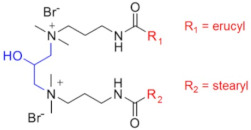	YS-18	- better temperature and salt tolerance than corresponding symmetric surfactants (stable under a share rate of 170 s^−1^ at 110 °C for 60 min); - gel breaking by reservoir brine and kerosene	no data	[65]
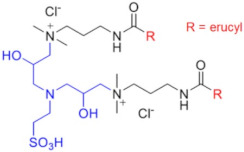	VES-S	- good salt tolerance (up to 8 wt % NaCl), fracturing fluid may be prepared with seawater; - sufficient viscoelastic properties and proppant-carrying capacity at high salinity; - gel breaking by kerosene	high temperature tolerance not studied in the work	[62]
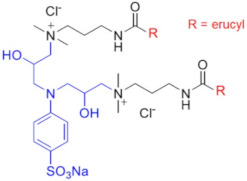	EDBS	- temperature, shear and salt resistance (stability under a share rate of 170 s^−1^ at 120 °C for 120 min in the 25% standard brine solution); - gel breaking by kerosene	no data	[63]
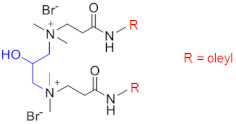	GLO	- sufficient viscoelastic properties; - shear resistance at 80 °C; - low proppant settling velocity; - low permeability and conductivity loss rates	high temperature tolerance not studied in the work	[64]
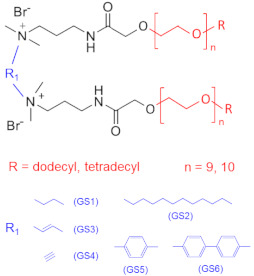	GS	- surfactant inhibits clay swelling; - sandstone retains its permeability at much higher level than for conventional clay stabilizers such as NaCl or KCl; - no increase in rock breakdown pressure	no influence of surfactants on the rheology of fluids reported	[66,67,68,69]

## Abbreviations

**Abbreviation**	**Expansion**
CMC	critical micelle cocentration
CMHPG	carboxymethyl hydroxypropyl guar
CNF	cellulose nanofibrils
CTAB	cetyltrimethylammonium bromide
EDAA	N-erucamidopropyl-N,N-dimethyl-N-allylammonium bromide
EHAC	erucyl bis-(hydroxyethyl)methylammonium chloride
EOR	enhanced oil recovery
HM PAA	hydrophobically modified polyacrylic acid
HM PAAm	hydrophobically modified polyacrylamide
HM HPG	hydrophobically modified hydroxypropyl guar
HPGNaSal	hydroxypropyl guarsodium salicylate
NP	nanoparticle
PSS	sodium polystyrenesulfonate
SDS	sodium dodecyl sulfate
SOMT	sodium oleoyl methyl taurate
TMPDA	N,N,N′,N′-tetramethyl-1,3-propanediamine
VES	viscoelastic surfactant
WLM	wormlike micelle
